# *Vital Signs:* Trends and Disparities in Childhood Vaccination Coverage by Vaccines for Children Program Eligibility — National Immunization Survey-Child, United States, 2012–2022

**DOI:** 10.15585/mmwr.mm7333e1

**Published:** 2024-08-22

**Authors:** Madeleine R. Valier, David Yankey, Laurie D. Elam-Evans, Michael Chen, Holly A. Hill, Yi Mu, Cassandra Pingali, Juan A. Gomez, Bayo C. Arthur, Tamara Surtees, Samuel B. Graitcer, Nicole F. Dowling, Shannon Stokley, Georgina Peacock, James A. Singleton

**Affiliations:** ^1^Immunization Services Division, National Center for Immunization and Respiratory Diseases, CDC; ^2^Oak Ridge Institute for Science and Education, Oak Ridge, Tennessee.

## Abstract

**Introduction:**

The Vaccines for Children (VFC) program was established in 1994 to provide recommended vaccines at no cost to eligible children and help ensure that all U.S. children are protected from life-threatening vaccine-preventable diseases.

**Methods:**

CDC analyzed data from the 2012–2022 National Immunization Survey-Child (NIS-Child) to assess trends in vaccination coverage with ≥1 dose of measles, mumps, and rubella vaccine (MMR), 2–3 doses of rotavirus vaccine, and a combined 7-vaccine series, by VFC program eligibility status, and to examine differences in coverage among VFC-eligible children by sociodemographic characteristics. VFC eligibility was defined as meeting at least one of the following criteria: 1) American Indian or Alaska Native; 2) insured by Medicaid, Indian Health Service (IHS), or uninsured; or 3) ever received at least one vaccination at an IHS-operated center, Tribal health center, or urban Indian health care facility.

**Results:**

Overall, approximately 52.2% of U.S. children were VFC eligible. Among VFC-eligible children born during 2011–2020, coverage by age 24 months was stable for ≥1 MMR dose (88.0%–89.9%) and the combined 7-vaccine series (61.4%–65.3%). Rotavirus vaccination coverage by age 8 months was 64.8%–71.1%, increasing by an average of 0.7 percentage points annually. Among all children born in 2020, coverage was 3.8 (≥1 MMR dose), 11.5 (2–3 doses of rotavirus vaccine), and 13.8 (combined 7-vaccine series) percentage points lower among VFC-eligible than among non–VFC-eligible children.

**Conclusions and implications for public health practice:**

Although the VFC program has played a vital role in increasing and maintaining high levels of childhood vaccination coverage for 30 years, gaps remain. Enhanced efforts must ensure that parents and guardians of VFC-eligible children are aware of, have confidence in, and are able to obtain all recommended vaccines for their children.

**Summary**

**What is already known about this topic?**

The Vaccines for Children (VFC) program covers the cost of vaccines for eligible children to help ensure that all U.S. children are protected from life-threatening vaccine-preventable diseases.

**What is added by this report?**

Among VFC-eligible children, coverage with measles, mumps, and rubella vaccine was high and stable during 2012 through 2022, but there is room for improvement to increase coverage with other routinely recommended vaccines. Among children born in 2020, vaccination coverage was 4–14 percentage points lower among children who were eligible versus non-eligible for the VFC program.

**What are the implications for public health practice?**

The VFC program plays a vital role in increasing and sustaining vaccination coverage. Increased efforts must promote awareness of, confidence in, and receipt of all recommended vaccines among those eligible for the VFC program.

## Introduction

Congress established the Vaccines for Children (VFC) program in 1994 to provide routine vaccines at no cost to eligible children. Since introduction of the VFC program, vaccination of children born during 1994–2023 will have prevented approximately 508 million illnesses and 1,129,000 deaths, saving nearly $2.7 trillion in societal costs (*1*). In 2023, VFC distributed approximately 74 million pediatric vaccine doses to participating health care provider locations (CDC, unpublished data, 2024). The VFC program is one of the nation’s primary health platforms created to promote health equity and improve the health of children.

VFC funds are allocated by the Centers for Medicare & Medicaid Services to CDC, and Medicaid providers can receive payment from Medicaid for vaccine administration services provided to Medicaid-eligible children.* CDC provides funding to 61 state, local, and territorial immunization programs to implement and oversee the VFC program (*2*). Persons aged ≤18 years who are Medicaid-eligible, uninsured, underinsured,^†^ or American Indian or Alaska Native (AI/AN)^§^ are eligible to receive vaccines from VFC program providers at no cost. This report 1) describes characteristics of children eligible for the VFC program; 2) examines trends in routine vaccination coverage among VFC-eligible children; and 3) identifies gaps in vaccination coverage among VFC-eligible children compared with children who are not VFC-eligible.

## Methods

### Data Collection

NIS-Child is a nationally representative household survey that monitors coverage with Advisory Committee on Immunization Practices (ACIP)–recommended vaccines among children aged 19–35 months in the 50 states, the District of Columbia, and some U.S. territories^¶^ using a random-digit–dial telephone sampling frame.** Parents and guardians (parents) of eligible children are interviewed to obtain child, maternal, and household information and to obtain consent to contact the child’s vaccine providers. With consent, parent-identified providers receive mailed immunization history questionnaires and are asked to provide information on vaccination types, doses, and dates administered and administrative data.

The overall household response rates^††^ for 2012–2022 NIS-Child surveys ranged from 21.1% to 42.5%. Adequate provider data^§§^ were available for 49.4% to 63.9% of children aged 19–35 months with a completed household interview, resulting in a sample size of 152,915 children.

### Data Analysis

VFC-eligible children were defined as meeting one of these criteria: 1) AI/AN; 2) enrolled in Medicaid or the Indian Health Service (IHS) or uninsured^¶¶^; or 3) ever received at least one vaccination at an IHS-operated center, Tribal health center, or urban Indian health care facility. Birth cohorts were constructed to assess coverage with ≥1 dose of MMR, 2–3 doses of rotavirus vaccine,*** the combined 7-vaccine series,^†††^ and other routinely recommended vaccines^§§§^ among children born during 2011–2020. Kaplan-Meier techniques were used to estimate vaccination coverage with all vaccines by age 24 months, with a few exceptions.^¶¶¶^ Percentage point differences in vaccination coverage between VFC-eligible and non–VFC-eligible children (i.e., coverage among VFC-eligible children minus coverage among non–VFC-eligible children) were analyzed using Z-tests to assess the gap in coverage by VFC program eligibility status. Weighted linear regression models assessed the average annual percentage point change (AAPPC) in vaccination coverage among children born during 2011–2020. Estimates of vaccination coverage with ≥1 dose MMR, rotavirus, and the combined 7-vaccine series were stratified by the child’s race and ethnicity, health insurance status, urbanicity,**** and household income. Analyses were conducted using SAS-callable SUDAAN (version 11.0.3, RTI International) with p<0.05 considered statistically significant. This activity was reviewed by CDC, deemed not research, and was conducted consistent with applicable federal law and CDC policy.^††††^

## Results

### Characteristics of Children Eligible for the VFC Program

Among children aged 19–35 months who were born during 2011–2020, 52.2% were VFC eligible (Table 1). Among VFC-eligible children born in 2020, 93.4% were Medicaid-insured, 7.4% were AI/AN, 43.7% lived in households with income below the federal poverty level, and 48.1% lived in a metropolitan statistical area (MSA) principal city. The proportion of VFC-eligible children who were uninsured decreased from 8.1% of those born in 2011 to 3.1% of those born in 2020.

**TABLE 1:** Characteristics of children aged 19–35 months born during 2011–2020, overall and by Vaccines for Children program eligibility* — National Immunization Survey-Child, United States, 2012–2022

Characteristic	Children born during 2011–2020				VFC-eligible children	
	Total		VFC-eligible	Non–VFC-eligible	Born in 2011	Born in 2020
	No.	% (95% CI)	% (95% CI)	% (95% CI)	% (95% CI)	% (95% CI)
**Overall**	**152,915**	**100.0 (—)**	**52.2 (51.7–52.7)**	**47.8 (47.3–48.3)**	**53.4 (51.8–55.0)**	**52.6 (50.7–54.4)**
**Health insurance status among VFC-eligible children**						
Any Medicaid insurance	**56,077**	**91.2 (90.8–91.6)**	91.2 (90.8–91.6)	NA	88.9 (87.3–90.3)	93.4 (92.0–94.6)
Indian Health Service	**1,199**	**1.0 (0.9–1.1)**	1.0 (0.9–1.1)	NA	1.0 (0.7–1.5)	0.8 (0.6–1.1)
No insurance	**4,083**	**5.7 (5.4–6.0)**	5.7 (5.4–6.0)	NA	8.1 (6.9–9.6)	3.1 (2.2–4.2)
Other insurance^†^	**1,926**	**2.1 (2.0–2.3)**	2.1 (2.0–2.3)	NA	1.9 (1.4–2.7)	2.7 (2.0–3.6)
**Health insurance status among non–VFC-eligible children**						
Private insurance only	**79,009**	**86.4 (85.9–86.8)**	NA	86.4 (85.9–86.8)	NA	NA
Other insurance^§^	**10,621**	**13.6 (13.2–14.1)**	NA	13.6 (13.2–14.1)	NA	NA
**Poverty status**						
At or above poverty level	**116,008**	**70.0 (69.5–70.5)**	46.5 (45.7–47.3)	94.5 (94.1–94.8)	37.8 (35.4–40.2)	56.3 (53.3–59.3)
Below poverty level	**31,612**	**30.0 (29.5–30.5)**	53.5 (52.7–54.3)	5.5 (5.2–5.9)	62.2 (59.8–64.6)	43.7 (40.7–46.7)
**Race and ethnicity^¶^**						
AI/AN, alone or combined with another race or ethnicity	**6,375**	**3.8 (3.6–4.0)**	7.3 (6.9–7.7)	NA	6.8 (5.8–8.1)	7.4 (6.2–8.7)
AI/AN only	**2,031**	**1.0 (0.9–1.1)**	1.9 (1.8–2.1)	NA	1.8 (1.4–2.2)	2.0 (1.6–2.6)
Asian only	**7,100**	**5.5 (5.2–5.7)**	3.9 (3.6–4.3)	7.2 (6.8–7.6)	3.4 (2.7–4.4)	4.1 (3.0–5.6)
Black or African American only	**12,230**	**13.0 (12.7–13.4)**	17.7 (17.2–18.3)	7.9 (7.5–8.3)	19.2 (17.4–21.2)	17.3 (15.5–19.3)
NH/OPI only	**689**	**0.3 (0.2–0.3)**	0.3 (0.2–0.4)	0.2 (0.2–0.3)	NA**	NA**
White only	**88,677**	**46.1 (45.6–46.6)**	30.8 (30.2–31.5)	62.7 (62.1–63.4)	30.5 (28.6–32.5)	31.6 (29.1–34.3)
Hispanic or Latino	**30,460**	**27.1 (26.6–27.6)**	37.6 (36.8–38.4)	15.7 (15.1–16.3)	37.4 (34.9–40.0)	36.7 (33.8–39.7)
Multiple races	**11,728**	**7.0 (6.8–7.3)**	7.7 (7.3–8.1)	6.3 (6.0–6.6)	7.2 (6.2–8.4)	7.9 (6.6–9.4)
**Urbanicity**						
MSA, principal city	**66,789**	**45.8 (45.3–46.3)**	47.8 (47.0–48.5)	43.7 (43.0–44.4)	47.7 (45.3–50.2)	48.1 (45.2–51.0)
MSA, nonprincipal city	**58,477**	**42.6 (42.1–43.1)**	37.9 (37.1–38.7)	47.7 (47.0–48.4)	36.2 (33.8–38.7)	37.5 (34.7–40.3)
Non-MSA	**27,649**	**11.6 (11.4–11.9)**	14.4 (13.9–14.8)	8.6 (8.3–8.9)	16.1 (14.7–17.5)	14.5 (12.7–16.4)

**Abbreviations:** AI/AN = American Indian or Alaska Native; CHIP = Children’s Health Insurance Program; MSA = metropolitan statistical area; NA = not applicable; NH/OPI = Native Hawaiian or other Pacific Islander; VFC = Vaccines for Children.

* Child identified as AI/AN; insured by Medicaid or Indian Health Service; uninsured; or received at least one vaccine at an Indian Health Service–operated center, Tribal health facility, or urban Indian health care facility.

^†^ Includes children identified as AI/AN with private insurance, CHIP, military, or another form of insurance, alone or in combination with another plan.

^§^ Includes children with CHIP, military, or other insurance, alone or in combination with private insurance.

^¶^ The child’s race and ethnicity was reported by their parent or guardian. Children identified as AI/AN, Asian, Black or African American, NH/OPI, White, or multiple races were reported by the parent or guardian as non-Hispanic. Children identified as having multiple races had more than one race category selected. Children identified as Hispanic or Latino might be of any race. Children identified as AI/AN, alone or in combination with another race or ethnicity, might not be mutually exclusive from other racial and ethnic groups shown.

** Estimate suppressed because it did not meet standards for data reliability (95% CI >20, relative SE >30, or sample size <30).

### Overall Vaccination Coverage Among Children Eligible for the VFC Program

Among VFC-eligible children born during 2011–2020, coverage by age 24 months with ≥1 dose of MMR and the combined 7-vaccine series was stable (88.0%–89.9% and 61.4%–65.3%, respectively) (Figure) (Table 2). Rotavirus vaccination coverage by age 8 months was 64.8%–71.1%, increasing on average by 0.7 percentage points annually. Among VFC-eligible children born in 2020, coverage with ≥1 dose of MMR, rotavirus vaccine, and the combined 7-vaccine series was 89.6%, 71.0%, and 61.4%, respectively.

**FIGURE:**
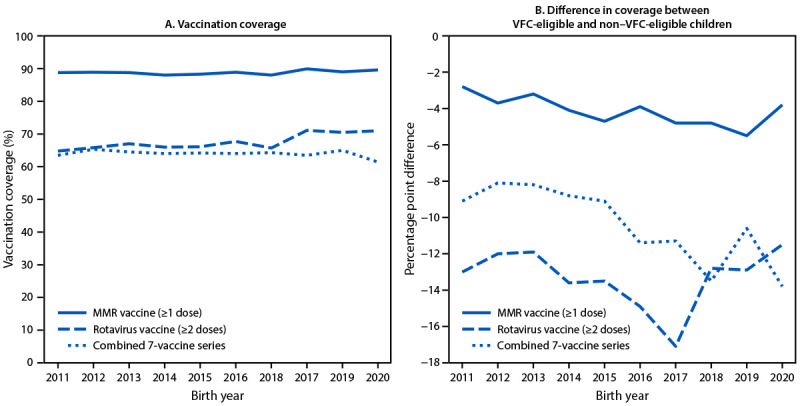
Coverage* with ≥1 dose of measles, mumps, and rubella vaccine,^†^ rotavirus vaccine,^§^ and the 7-vaccine series^¶^ among children eligible** for the Vaccines for Children program (A) and difference in vaccination coverage between program-eligible and nonprogram-eligible children^††^ born during 2011–2020 (B) — National Immunization Survey-Child, United States, 2012–2022 **Abbreviations:** AI/AN = American Indian or Alaska Native; MMR = measles, mumps, and rubella vaccine; VFC = Vaccines for Children. * Coverage with ≥1 dose of MMR and the combined 7-vaccine series assessed before the day the child turns 24 months. Rotavirus vaccination coverage assessed by age 8 months, 0 days. The Kaplan-Meier method was used to estimate vaccination coverage to account for children whose vaccination history was ascertained before age 24 months. ^†^ Includes children who might have received measles, mumps, rubella, and varicella combination vaccine. ^§^ At least two doses of Rotarix monovalent rotavirus vaccine, or ≥3 doses of RotaTeq pentavalent rotavirus vaccine. If any dose in the series is either RotaTeq or unknown, it was assumed a 3-dose series was needed. Maximum age for receipt of the final dose is age 8 mos, 0 days. **^¶^** The combined 7-vaccine series (4:3:1:3*:3:1:4) includes ≥4 doses of diphtheria and tetanus toxoids and acellular pertussis vaccine, ≥3 doses of poliovirus vaccine, ≥1 dose of measles-containing vaccine, the full series of *Haemophilus influenzae* type b conjugate vaccine (≥3 or ≥4 doses, depending on product type), ≥3 doses of hepatitis B vaccine, ≥1 dose of varicella vaccine, and ≥4 doses of pneumococcal conjugate vaccine. ** Child is identified as AI/AN; insured by Medicaid or Indian Health Service; uninsured; or received any vaccines at an Indian Health Service–operated center, Tribal health facility, or urban Indian health care facility. ^††^ Defined as coverage among VFC-eligible children − coverage among non–VFC eligible children.

**TABLE 2:** Trends in vaccination coverage* with ≥1 dose measles, mumps, and rubella vaccine,^†^ rotavirus vaccine,^§^ and the combined 7-vaccine series^¶^ among children eligible** for the Vaccines for Children program and difference in coverage between program-eligible and nonprogram-eligible children born during 2011–2020 — National Immunization Survey-Child, United States, 2012–2022

Birth year	Vaccine measure					
	MMR^†^ (≥1 dose)		Rotavirus^§^ (received by age 8 mos, 0 days)		Combined 7-vaccine series^¶^	
	Coverage % (95% CI)	Percentage point difference (95% CI)	Coverage % (95% CI)	Percentage point difference (95% CI)	Coverage % (95% CI)	Percentage point difference (95% CI)
**2011**	**88.8 (87.1 to 90.4)**	**–2.8 (–4.9 to –0.7)^††^**	**64.8 (62.3 to 67.1)**	**–13.0 (–16.0 to –10.0)^††^**	**63.5 (61.1 to 65.9)**	**–9.1 (–12.2 to –5.9)^††^**
2012	88.9 (87.4 to 90.2)	–3.7 (–5.4 to –2.0)^††^	65.8 (63.5 to 67.9)	–12.0 (–15.0 to –9.1)^††^	65.3 (63.0 to 67.5)	–8.1 (–11.1 to –5.0)^††^
2013	88.8 (87.3 to 90.2)	–3.2 (–5.1 to –1.2)^††^	67.0 (64.8 to 69.2)	–11.9 (–14.6 to –9.1)^††^	64.5 (62.2 to 66.7)	–8.2 (–11.2 to –5.3)^††^
2014	88.0 (86.2 to 89.7)	–4.1 (–6.3 to –1.8)^††^	66.0 (63.5 to 68.4)	–13.6 (–16.8 to –10.4)^††^	64.0 (61.6 to 66.4)	–8.8 (–12.2 to –5.4)^††^
2015	88.3 (86.8 to 89.8)	–4.7 (–6.5 to –2.9)^††^	66.1 (63.6 to 68.6)	–13.5 (–16.7 to –10.3)^††^	64.2 (61.7 to 66.7)	–9.1 (–12.3 to –5.8)^††^
2016	88.9 (87.2 to 90.4)	–3.9 (–5.8 to –2.0)^††^	67.7 (65.1 to 70.2)	–14.9 (–17.9 to –11.9)^††^	64.0 (61.4 to 66.7)	–11.4 (–14.7 to –8.1)^††^
2017	88.0 (86.5 to 89.5)	–4.8 (–6.8 to –2.7)^††^	65.7 (63.5 to 67.8)	–17.1 (–19.7 to –14.4)^††^	64.3 (62.1 to 66.6)	–11.3 (–14.2 to –8.4)^††^
2018	89.9 (88.5 to 91.2)	–4.8 (–6.4 to –3.3)^††^	71.1 (69.1 to 73.0)	–12.8 (–15.2 to –10.4)^††^	63.5 (61.4 to 65.6)	–13.5 (–16.2 to –10.9)^††^
2019	89.0 (87.6 to 90.4)	–5.5 (–7.2 to –3.9)^††^	70.5 (68.5 to 72.5)	–12.9 (–15.4 to –10.5)^††^	65.0 (62.8 to 67.2)	–10.6 (–13.3 to –7.8)^††^
2020^§§^	89.6 (87.7 to 91.3)	–3.8 (–6.0 to –1.6)^††^	71.0 (68.3 to 73.5)	–11.5 (–14.8 to –8.3)^††^	61.4 (58.4 to 64.4)	–13.8 (–17.6 to –10.0)^††^
AAPPC^¶¶^	0.1 (–0.1 to 0.2)	–0.2 (–0.4 to –0.1)***	0.7 (0.3 to 1.1)***	–0.1 (–0.5 to 0.4)	–0.1 (–0.3 to 0.1)	–0.6 (–0.9 to –0.3)***

**Abbreviations:** AAPPC = average annual percentage point change in coverage; AI/AN = American Indian or Alaska Native; MMR = measles, mumps, and rubella vaccine; VFC = Vaccines for Children.

* Coverage with ≥1 dose MMR and the combined 7-vaccine series were assessed by age 24 months (before the day the child turns 24 months). Rotavirus vaccine series was assessed by age 8 months, 0 days. The Kaplan-Meier method was used to estimate vaccination coverage to account for children whose vaccination history was ascertained before age 24 months.

^†^ Includes children who might have received measles, mumps, rubella, and varicella combination vaccine.

^§^ Two or more doses of Rotarix monovalent rotavirus vaccine, or ≥3 doses of RotaTeq pentavalent rotavirus vaccine. (If any dose in the series is either RotaTeq or unknown, it was assumed a 3-dose series was needed. The maximum age for receipt of the final rotavirus vaccine dose is 8 months, 0 days.

^¶^ The combined 7-vaccine series (4:3:1:3*:3:1:4) includes ≥4 doses of diphtheria and tetanus toxoids and acellular pertussis vaccine, ≥3 doses of poliovirus vaccine, ≥1 dose of measles-containing vaccine, the full series of *Haemophilus influenzae* type b conjugate vaccine (≥3 or ≥4 doses, depending on product type), ≥3 doses of hepatitis B vaccine, ≥1 dose of varicella vaccine, and ≥4 doses of pneumococcal conjugate vaccine.

** Child is identified as AI/AN; insured by Medicaid or Indian Health Service; uninsured; or received at least one vaccine at an Indian Health Service–operated center, Tribal health facility, or urban Indian health care facility.

^††^ Statistically significant (p<0.05) percentage point difference in coverage (VFC-eligible − non–VFC-eligible).

^§§^ Data for the 2020 birth year are considered preliminary and are from survey years 2021 and 2022. Data from survey year 2023 were not available in time to include in this report.

^¶¶^ Slope of line created by fitting a linear regression model to the coverage estimates from birth years 2011–2020.

*** AAPPC is statistically significantly different from zero (p<0.05).

Among the vaccines included in the combined 7-vaccine series, coverage among VFC-eligible children born in 2020 was approximately 90% for first doses of vaccines (≥1 dose of varicella vaccine and ≥1 dose of MMR^§§§§^) and for series administered earlier in life (≥3 doses of poliovirus vaccine and ≥3 doses of hepatitis B vaccine) (Supplementary Table, https://stacks.cdc.gov/view/cdc/159296). Coverage was 73.6%–76.7% with series requiring multiple doses by age 24 months, with some doses recommended after age 12 months (i.e., ≥4 doses of diphtheria, tetanus toxoids, and acellular pertussis vaccine; ≥4 doses pneumococcal conjugate vaccine; and the full series of *Haemophilus influenzae* type b conjugate vaccine).

### Vaccination Coverage Among VFC-Eligible Children Born in 2020 by Selected Characteristics

Among VFC-eligible children born in 2020, coverage with ≥1 dose of MMR, rotavirus vaccine, and the combined 7-vaccine series among those who were uninsured was 18.9–34.7 percentage points lower than that among Medicaid-insured children (Table 3). Compared with coverage among children living at or above the poverty level, coverage with rotavirus vaccine and the combined 7-vaccine series among those living below the poverty level was 9.3–9.9 percentage points lower. By race and ethnicity, rotavirus vaccination coverage among AI/AN and Hispanic or Latino children was 6.9–8.9 percentage points higher than that among non-Hispanic White (White) children.

**TABLE 3:** Vaccination coverage* among children born in 2020^†^ and eligible^§^ for the Vaccines for Children program, by sociodemographic characteristics, and difference in coverage compared with children not eligible for the Vaccines for Children program — National Immunization Survey-Child, United States, 2021–2022

Characteristic	Vaccine measure					
	MMR^¶^ (≥1 dose)		Rotavirus (received by age 8 mos, 0 days)**		Combined 7-vaccine series^††^	
	VFC-eligible children coverage % (95% CI)	PP difference^§§ ^(95% CI)	VFC-eligible children coverage % (95% CI)	PP difference^§§ ^(95% CI)	VFC-eligible children coverage % (95% CI)	PP difference^§§ ^(95% CI)
**Overall vaccination coverage**	**89.6 (87.7 to 91.3)**	**–3.8 (–6.0 to –1.6)^¶¶^**	**71.0 (68.3 to 73.5)**	**–11.5 (–14.8 to –8.3)^¶¶^**	**61.4 (58.4 to 64.4)**	**–13.8 (–17.6 to –10.0)^¶¶^**
**Health insurance status**						
Any Medicaid insurance (Ref)	90.1 (88.2 to 91.9)	NA	71.8 (69.0 to 74.4)	NA	61.8 (58.6 to 64.9)	NA
Indian Health Service	92.6 (81.2 to 98.3)	NA	78.5 (62.5 to 88.9)***	NA	71.6 (57.8 to 84.1)***	NA
No insurance	71.2 (56.5 to 84.5)***^,†††^	NA	37.1 (24.6 to 51.6)***^,†††^	NA	39.3 (25.5 to 57.1)***^,†††^	NA
Other insurance^§§§^	89.1 (80.1 to 95.2)	NA	78.1 (64.2 to 87.6)***	NA	69.6 (56.7 to 81.6)***	NA
**Poverty status**						
At or above poverty level (Ref)	88.9 (86.0 to 91.4)	–4.6 (–7.6 to –1.6)^¶¶^	76.1 (72.7 to 79.1)	–7.7 (–11.5 to –4.0)^¶¶^	65.3 (61.5 to 69.2)	–11.2 (–15.7 to –6.8)^¶¶^
Below poverty level	89.5 (86.7 to 92.0)	0 (–8.0 to 7.9)	66.2 (61.6 to 70.4)^†††^	6.0 (–10.0 to 21.9)***	56.0 (51.1 to 61.0)^†††^	–2.6 (–20.1 to 14.9)***
**Race and ethnicity^¶¶¶^**						
AI/AN, alone or combined with another race or ethnicity****	92.0 (87.6 to 95.2)	NA	75.5 (68.5 to 81.4)^†††^	NA	67.7 (59.4 to 75.8)	NA
AI/AN only	89.1 (81.3 to 94.6)	NA	69.0 (56.8 to 79.0)***	NA	56.7 (44.6 to 69.5)***	NA
Asian only	90.3 (77.8 to 97.4)	–2.8 (–14.2 to 8.5)***	79.3 (64.3 to 89.1)***	–4.7 (–18.9 to 9.5)***	62.7 (46.8 to 78.7)***	–15.7 (–33.8 to 2.4)***
Black or African American only	88.9 (84.2 to 92.7)	0.8 (–6.0 to 7.5)	71.7 (66.3 to 76.5)	–7.6 (–15.9 to 0.7)	61.8 (55.9 to 67.8)	–4.2 (–14.9 to 6.6)
White only (Ref)	88.3 (85.1 to 91.1)	–6.2 (–9.4 to –3.0)^¶¶^	66.6 (61.9 to 71.1)	–17.9 (–22.9 to –12.9)^¶¶^	60.3 (55.6 to 65.0)	–17.1 (–22.4 to –11.8)^¶¶^
Hispanic or Latino	91.7 (88.8 to 94.1)	–1.2 (–6.2 to 3.8)	73.5 (68.5 to 78.0)^†††^	–6.1 (–14.2 to 2.0)	62.0 (56.1 to 67.9)	–9.7 (–18.9 to –0.5)^¶¶^
Multiple races	86.6 (75.2 to 94.5)	–6.6 (–17.4 to 4.2)***	71.3 (61.4 to 79.5)	–5.5 (–17.5 to 6.6)***	63.0 (53.0 to 73.0)	–11.0 (–24.1 to 2.0)***
**Urbanicity**						
MSA, principal city (Ref)	89.4 (86.7 to 91.7)	–3.3 (–6.4 to –0.3)^¶¶^	72.2 (68.4 to 75.8)	–12.4 (–16.9 to –8.0)^¶¶^	62.2 (58.0 to 66.5)	–12.8 (–18.3 to –7.3)^¶¶^
MSA, nonprincipal city	90.4 (87.6 to 92.9)	–4.1 (–7.2 to –1.0)^¶¶^	69.9 (65.3 to 74.2)	–11.6 (–16.9 to –6.3)^¶¶^	61.4 (56.1 to 66.7)	–15.4 (–21.6 to –9.3)^¶¶^
Non-MSA	88.4 (82.2 to 93.2)	–0.7 (–10.1 to 8.6)	69.4 (62.2 to 75.8)	–6.6 (–17.1 to 3.9) ***	59.1 (52.2 to 66.1)	–6.0 (–16.5 to 4.6)***

**Abbreviations:** AI/AN = American Indian or Alaska Native; CHIP = Children’s Health Insurance Program; MMR = measles, mumps, and rubella vaccine; MSA = metropolitan statistical area; NA = not applicable; PP = percentage point; Ref = referent group; VFC = Vaccines for Children.

* Coverage with ≥1 dose MMR and the combined 7-vaccine series were assessed by age 24 months (before the day the child turns 24 months). Rotavirus vaccine series was assessed by age 8 months 0 days. The Kaplan-Meier method was used to estimate vaccination coverage to account for children whose vaccination history was ascertained before age 24 months.

^†^ Data for the 2020 birth year are considered preliminary and are from survey years 2021 and 2022. Data from survey year 2023 were not available in time to include in this report.

^§^ Child is identified as AI/AN; insured by Medicaid or Indian Health Service; uninsured; or received at least one vaccine at an Indian Health Service–operated center, Tribal health facility, or urban Indian health care facility.

^¶^ Includes children who might have received measles, mumps, rubella, and varicella combination vaccine.

** Includes ≥2 doses of Rotarix monovalent rotavirus vaccine, or ≥3 doses of RotaTeq pentavalent rotavirus vaccine. If any dose in the series is either RotaTeq or unknown, it was assumed a 3-dose series was needed. The maximum age for the final rotavirus dose is 8 months, 0 days.

^††^ The combined 7-vaccine series (4:3:1:3*:3:1:4) includes ≥4 doses of diphtheria and tetanus toxoids and acellular pertussis vaccine, ≥3 doses of poliovirus vaccine, ≥1 dose of measles-containing vaccine, the full series of *Haemophilus influenzae* type b conjugate vaccine (≥3 or ≥4 doses, depending on product type), ≥3 doses of hepatitis B vaccine, ≥1 dose of varicella vaccine, and ≥4 doses of pneumococcal conjugate vaccine.

^§§^ PP difference in coverage = coverage among VFC-eligible children − coverage among non–VFC-eligible children.

^¶¶^ PP difference in coverage is statistically significant (p<0.05).

*** Estimates with 95% CIs >20 might not be reliable.

^†††^ Statistically significant difference in coverage (p<0.05) compared with Ref.

^§§§^ Includes children identified as AI/AN with private insurance, CHIP, military, or another form of insurance, alone or in combination with another plan.

^¶¶¶^ The child’s race and ethnicity was reported by their parent or guardian. Children identified as AI/AN, Asian, Black or African American, Native Hawaiian or other Pacific Islander, White, or multiple races were reported by the parent or guardian as non-Hispanic. Children identified as having multiple races had more than one race category selected. Children identified as Hispanic or Latino might be of any race. Children identified as AI/AN, alone or in combination with another race or ethnicity, might not be mutually exclusive from other racial and ethnic groups shown. Estimates for Native Hawaiian or other Pacific Islander children were suppressed because of small sample size.

**** Comparisons by race and ethnicity for AI/AN children, in combination with another race or ethnicity, and White children were possible as these racial and ethnic groups were mutually exclusive among children born in 2020.

### Comparison of Vaccination Coverage Among VFC-Eligible and Non–VFC-Eligible Children

Among all children born during 2011–2020, coverage with ≥1 dose of MMR, rotavirus vaccine, and the combined 7-vaccine series was lower among VFC-eligible children than among non–VFC-eligible children (Figure) (Table 2). During this period, the gap in coverage between VFC-eligible and non–VFC-eligible children increased for ≥1 dose of MMR (AAPPC = −0.2) and the combined 7-vaccine series (AAPPC = −0.6). Among children born in 2020, coverage with ≥1 dose of MMR, rotavirus vaccine, and the combined 7-vaccine series was 3.8, 11.5, and 13.8 percentage points, respectively, lower among VFC-eligible than among non–VFC-eligible children.

Among children born in 2020, all three vaccination coverage measures were lower among VFC-eligible children than among non–VFC-eligible children who were 1) White (−17.9 to −6.2 percentage points), 2) living at or above the poverty level (−11.2 to −4.6 percentage points), and 3) living in MSA principal cities (−12.8 to −3.3 percentage points) and MSA nonprincipal cities (−15.4 to −4.1 percentage points) (Table 3). Statistically significant gaps in coverage by sociodemographic characteristics were narrower for ≥1 dose of MMR (−6.2 to −3.3 percentage points) and wider for rotavirus vaccine (−17.9 to −7.7 percentage points) and the combined 7-vaccine series (−17.1 to −9.7 percentage points).

## Discussion

More than one half of U.S. children (52.6%) born in 2020 were eligible for the VFC program, underscoring the vast scope of this program 30 years after it was enacted into law. Coverage among VFC-eligible children born during 2011–2020 with ≥1 dose of MMR remained high and stable, indicating that efforts to achieve and maintain measles elimination status in the United States have been supported through the VFC program. No differences in ≥1-dose MMR coverage among VFC-eligible children born in 2020 were found by race and ethnicity, poverty status, and urban-rural residency, demonstrating continued success in providing equitable access to vaccination through the VFC program (*3*). Increased coverage with rotavirus vaccine among VFC-eligible children born during 2011–2020 signals progress toward achieving high coverage with all routinely recommended immunizations.

Children born during 2018–2020 might have experienced health care disruptions resulting from the COVID-19 pandemic. However, previous analyses^¶¶¶¶^ found no differences in overall vaccination coverage by age 24 months among children who were due for vaccination before the pandemic compared with those who were due for vaccination during the COVID-19 pandemic, including among children who were Medicaid-insured, uninsured, or AI/AN (*4**,**5*).

Coverage with the combined 7-vaccine series was 61.4% among VFC-eligible children born in 2020, highlighting room for improvement. By individual vaccine measures, coverage with first doses of vaccines and series administered earlier in life was high but was lower for multidose series vaccines, with additional doses administered at age >12 months. These patterns suggest potential barriers associated with receiving multidose series and for vaccinating VFC-eligible children during the second year of life. Provider reminder-recall systems and simultaneous administration of childhood vaccines at well-child visits have been established as effective strategies that can reduce missed vaccination opportunities and increase coverage (*6**,**7*).

Additional opportunities to improve coverage were identified among certain sociodemographic groups. Coverage was lower among uninsured children than among Medicaid-insured children, consistent with findings on vaccination coverage among uninsured adolescents and adults (*8**,**9*). Uninsured children are more likely to live in households with incomes below the poverty level, to have had no provider health care visits in the past year, and to be less likely to complete multidose vaccination series (*8*,*10*,*11*). The proportion of uninsured children was small and decreased from approximately 8.1% in 2011 to 3.1% in 2020. Efforts to further reduce the proportion of uninsured children, including increasing access to Medicaid, can facilitate connection to the health care system (*12*) and subsequently increase vaccination coverage (*13*).

Lower coverage with rotavirus vaccine and the combined 7-vaccine series was found among VFC-eligible children living below the poverty level compared with coverage among VFC-eligible children living at or above poverty. Although the VFC program provides vaccine at no cost, office visit fees or fees for nonvaccine services received during the visit (*2*) beyond vaccination cost might present potential barriers for low-income households, in addition to other barriers involving health care providers, parents, and the health care delivery system (*14*,*15*). Establishment of a place to receive ongoing routine care has been associated with increased likelihood of children in low-income households and VFC-eligible children being up to date with recommended vaccines (*14*,*16*).

Compared with coverage among non–VFC-eligible children, coverage overall was lower among VFC-eligible children, consistent with an earlier analysis of vaccination coverage among VFC-eligible children (*17*). High, yet lower ≥1-dose MMR coverage among VFC-eligible children compared with non–VFC-eligible children is concerning, because small pockets of low coverage have resulted in measles outbreaks (*18*,*19*). Despite improvements in rotavirus vaccination coverage among VFC-eligible children, coverage in this group was significantly lower than coverage among non–VFC-eligible children. Increased efforts are needed to ensure that parents of VFC-eligible children are aware of, have confidence in, and are able to obtain all recommended vaccines for their children.

### Limitations

The findings in this report are subject to at least six limitations. First, overall household response rates for NIS-Child were low (range = 21.1%–42.5%), and 49.4%–63.9% of children with completed household interviews had provider-reported vaccination records. Selection bias resulting from low household response rates might have occurred if the characteristics of participants and nonparticipants differed systematically. Data were weighted to account for nonresponse and households without telephones, but some bias might remain, which could affect the generalizability of results. Second, total survey error assessments***** indicate that NIS-Child data might underestimate actual coverage with some vaccines; thus, actual vaccination coverage might be higher than reported. Third, the definition of VFC eligibility status used for this study might have resulted in underestimation of the actual VFC-eligible population because the operationalized definition includes Medicaid-enrolled but not Medicaid-eligible children. If Medicaid-eligible children differ from those who are Medicaid-enrolled, comparisons by VFC eligibility status could be higher or lower. Fourth, underinsured children who received vaccines at a federally qualified health center, a rural health center, or a deputized provider^†††††^ were excluded from the VFC-eligible group because of difficulty ascertaining information on the underinsured through NIS. This exclusion could result in potential misclassification of underinsured children as non–VFC-eligible. Fifth, health insurance status was determined at time of interview and might have varied during the child's vaccination history, which could result in misclassification of VFC eligibility status. Sixth, this study was cross-sectional; therefore, underlying causes of observed differences in coverage over time or by VFC eligibility status could not be determined.

### Implications for Public Health Practice

The VFC program has supported high and increasing childhood vaccination coverage for 30 years and is one of public health’s primary platforms for equity and ensuring that all children can access vaccines. Despite successes, the need to increase coverage with all routine vaccines and to reach children living in lower-income households and who lack insurance continues. Health care provider interventions to improve coverage include encouraging providers to make strong vaccine recommendations for their patients, strengthening family-provider relationships, providing parental education about vaccine benefits, using reminder-recall systems, reducing missed opportunities for vaccination, offering simultaneous administration of childhood vaccines, and administering catch-up vaccinations to all inadequately vaccinated children (*6*,*7*).

Enactment of the VFC program 30 years ago was a historic step in improving children’s lives and advancing public health. The data presented in this report demonstrate long-term program results for multiple birth cohorts of children. As new vaccines are added and immunization schedules become increasingly complex, maintenance and evolution of the VFC program could help sustain and further increase vaccination coverage. Realizing this will require efforts to promote participation in the VFC program by providers serving VFC-eligible children. CDC encourages providers to assess vaccination needs for all children at every health care visit and strongly recommend needed vaccines, and address patient barriers and promote confidence in vaccination.

## Footnotes

* https://www.ssa.gov/OP_Home/ssact/title19/1928.htm

† Children categorized as underinsured because their health insurance plans do not include coverage for recommended vaccinations are eligible to receive VFC vaccines only at Federally Qualified Health Centers (FQHCs), Rural Health Clinics (RHCs), or under an approved deputization provider location agreement. https://www.cdc.gov/vaccines-for-children/vfc-information-for-parents/index.html

§ As defined by the Indian Health Care Improvement Act.

¶ Certain local areas that receive federal immunization funds under Section 317 of the Public Health Service Act are sampled separately in NIS. Those areas include Chicago, Illinois; New York, New York; Philadelphia County, Pennsylvania; Bexar County, Texas; and Houston, Texas. Two territories were sampled separately in 2022: Guam and Puerto Rico. National estimates in this report exclude U.S. territories.

** NIS-Child surveys from 2011–2017 were conducted using a dual-frame design with both mobile and landline sampling frames. NIS-Child surveys from 2018–2022 were conducted using a single-frame mobile telephone design.

†† The Council of American Survey Research Organizations response rate is the product of three other rates: 1) the resolution rate (the proportion of telephone numbers that can be identified as either for business or residence), 2) the screening rate (the proportion of qualified households that complete the screening process), and 3) the cooperation rate (the proportion of contacted eligible households for which a completed interview is obtained).

§§ Beginning with the 2012 NIS-Child, all children with any provider-reported vaccination data were considered to have adequate provider data. https://ftp.cdc.gov/pub/Health_Statistics/NCHS/Dataset_Documentation/NIS/NISPUF14_DUG.PDF

¶¶ Children not covered by any kind of health insurance are classified as uninsured. https://www.cdc.gov/vaccines-for-children/downloads/operations-guide-508.pdf

*** Includes ≥2 doses of Rotarix monovalent rotavirus vaccine or ≥3 doses of RotaTeq pentavalent rotavirus vaccine. If any dose in the series is either RotaTeq or unknown, it was assumed a 3-dose series was needed. The maximum age for the final rotavirus dose is 8 months, 0 days.

††† The combined 7-vaccine series (4:3:1:3*:3:1:4) includes ≥4 doses of diphtheria and tetanus toxoids and acellular pertussis vaccine (DTaP); ≥3 doses of poliovirus vaccine; ≥1 dose of measles-containing vaccine; ≥3 or ≥4 doses of *Haemophilus influenzae* type b conjugate vaccine (Hib) (depending upon product type); ≥3 doses of hepatitis B vaccine (HepB); ≥1 dose of varicella vaccine; and ≥4 doses of pneumococcal conjugate vaccine (PCV).

§§§ Coverage was also assessed for ≥4 doses of DTaP; ≥3 doses of poliovirus vaccine; ≥3 or ≥4 doses of Hib (depending upon product type); the HepB birth dose; ≥3 doses of HepB; ≥1 dose of varicella vaccine; ≥4 doses of PCV; ≥1 dose of hepatitis A vaccine (HepA); and ≥2 doses of influenza vaccine.

¶¶¶ Exceptions include coverage with the HepB birth dose, which was measured as the proportion of children who received a dose of HepB by age 3 days and rotavirus vaccine, assessed at age 8 months, 0 days.

**** MSA status was determined based on household-reported city and county of residence and was grouped into three categories: MSA principal city, MSA nonprincipal city, and non-MSA. MSAs and principal cities were as defined by the U.S. Census Bureau (https://www.census.gov/programs-surveys/metro-micro.html). Non-MSA areas include urban populations not located within an MSA and completely rural areas.

†††† 45 C.F.R. part 46.102(l)(2), 21 C.F.R. part 56; 42 U.S.C. Sect. 241(d); 5 U.S.C. Sect. 552a; 44 U.S.C. Sect. 3501 et seq.

§§§§ MMR and varicella vaccines are 2-dose vaccination series, with first doses administered at age 12–15 months, and second doses administered at age 4–6 years.

¶¶¶¶
https://www.cdc.gov/vaccines/imz-managers/coverage/childvaxview/pubs-presentations/nis-child-pandemic-effects-2018-2021.html

*****
https://www.cdc.gov/vaccines/imz-managers/coverage/childvaxview/pubs-presentations/downloads/Error-Profile-for-the-2022-NIS-Child_2023-10-04.pdf

††††† Deputization is the formal extension of VFC authority to provide VFC vaccines to eligible underinsured children from a participating FQHC or RHC to another VFC-enrolled provider. Under this arrangement, the deputizing FQHC or RHC retains its full scope of authority as a VFC provider while extending the authority to deputized VFC providers to immunize underinsured children with VFC vaccines.
